# Multi-Target Coordinated Search Algorithm for Swarm Robotics Considering Practical Constraints

**DOI:** 10.3389/fnbot.2021.753052

**Published:** 2021-12-06

**Authors:** You Zhou, Anhua Chen, Xinjie He, Xiaohui Bian

**Affiliations:** ^1^Department of Mechanical and Electrical Engineering, Hunan University of Science and Technology, Xiangtan, China; ^2^Intelligent Manufacturing College, Hunan Vocational Institute of Technology, Xiangtan, China; ^3^Department of Information and Electrical Engineering, Hunan University of Science and Technology, Xiangtan, China

**Keywords:** multi-target search, swarm robots, roaming search, coordinated search, simplified virtual force model, distributed neighborhood communication

## Abstract

In order to deal with the multi-target search problems for swarm robots in unknown complex environments, a multi-target coordinated search algorithm for swarm robots considering practical constraints is proposed in this paper. Firstly, according to the target detection situation of swarm robots, an ideal search algorithm framework combining the strategy of roaming search and coordinated search is established. Secondly, based on the framework of the multi-target search algorithm, a simplified virtual force model is combined, which effectively overcomes the real-time obstacle avoidance problem in the target search of swarm robots. Finally, in order to solve the distributed communication problem in the multi-target search of swarm robots, a distributed neighborhood communication mechanism based on a time-varying characteristic swarm with a restricted random line of sight is proposed, and which is combined with the multi-target search framework. For the swarm robot kinematics, obstacle avoidance, and communication constraints of swarm robots, the proposed multi-target search strategy is more stable, efficient, and practical than the previous methods. The effectiveness of this proposed method is verified by numerical simulations.

## Introduction

Inspired by the group behavior of social insects such as ants and bees, the concept of swarm intelligence is put forward by scholars (Bonabeau, 1999), which is defined as the collective intelligence emerging from a group of simple agents. The swarm robot system (Doty and Van Aken, 2002) is a typical artificial swarm intelligence system, which consists of a large number of homogeneous autonomous robots with a simple structure. By the coordination and cooperation of robots with limited individual capabilities under a specific mechanism, the system can present intelligent behavior and complete relatively complex tasks.

The common research contents of swarm robot systems include target search (Alfeo et al., 2019; Booth et al., 2020), task assignment (Liang et al., 2018), cluster avoidance (Khan et al., 2019), path planning (Ryan, 2008; Luo et al., 2017), and cluster formation (Anonymous, 1993; Alsamman, 2011). In this paper, the target search problem of swarm robots in unknown complex environments is mainly studied, such as forest fire detection (Yao et al., 2018; Marzaeva, 2019), toxic gas leak detection (Zhang et al., 2010; Moshayedi and Gharpure, 2013), search and rescue of missing personnel (Goodrich et al., 2009; Kamegawa et al., 2020), military target detection (Ha and Cho, 2018; Jiong et al., 2019) and so on. In order to solve this type of search problem, there are mainly composed of two main categories of design strategies, namely, behavior-based search and learning-based search (Cizek and Faigl, 2019; Berscheid et al., 2020; Suzuki et al., 2020), and this article mainly discusses the former.

According to the number of search targets, searches can be divided into single-target searches and multi-target searches. When the swarm robot system is applied to single-target search, it is necessary to pay attention to the cooperation mechanism between individual robots. Gudise (2004) proposed an extended particle swarm optimization (EPSO) algorithm, which was successfully applied to single-target searches. Ducatelle et al. (2011) used the local wireless network communication strategy to strengthen the communication ability between robots and enhance the robustness of the swarm robot system. Majid and Arshad (2017) mainly focused on the performance indicators in the EPSO coordinated search algorithm such as trajectory smoothness, search success rate, and search time, and studied the impact of the inertial weight on the search performance of swarm robots. Tang et al. (2020) proposed an improved adaptive bat algorithm (IABA) search algorithm by focussing on the problem of obstacle avoidance and improving the performance of the algorithm in the single-target search process of swarm robots. Aiming at the distributed communication problem in the single-target search process of swarm robots, Yang et al. (2019) proposed a time-varying characteristics swarm of visual limited (V-TVCS) model.

However, when the swarm robot system is applied to the actual neighborhood search, the number of search targets is more than one. Therefore, how to set up a multi-target search algorithm considering the actual search environment is the focus of scholars at home and abroad. Manic (2009) proposed a multi-target task allocation model with response threshold (TRT) to realize self-organizing task allocation, and then robots with the same objective task used the EPSO algorithm for coordinated search. Zhang and Xue (2014) proposed a dynamic task division strategy with closed-loop adjustment for the problem of uneven subgroup size of the TRT model. Xinjie (2020) established a simplified virtual force model (SVFM) for the unknown and complex environment, and successfully solved the obstacle avoidance problem in the multi-target search process. Zhang and Xue (2015) proposed the strategies of competition and cooperation and cooperation for the problem of subgroup interaction in parallel search. Jie (2019) proposed a probabilistic finite state machine search framework for the multi-target search problem of swarm robots. Xinjie (2020) extended the two-dimensional SVFM (2D-SVFM) to 3D-space, and successfully implemented this type of search method to achieve multi-target search in the Unmanned Aerial Vehicle (UAV) cluster system.

Based on the above literature analysis, the above methods can be applied to specific target search scenarios, but there are the following problems. First of all, there is no standard multi-target search algorithm framework in these methods. Most of the algorithms' settings are only suitable for searching for a specific number of targets, not for searching for any number of targets. Secondly, most of the algorithms only start to study a specific performance index of swarm robots, and do not consider the algorithm performance, obstacle avoidance, and swarm communication problem of swarm robots in actual search scenarios at the same time.

Aiming at the static multi-target search problem of swarm robots in unknown complex environments, a multi-target coordinated search algorithm for swarm robots considering practical constraints (MSRCPC) is proposed in this paper. The main work of this paper is as follows. First, based on the mechanism of finite state machines, an ideal multi-target search framework for swarm robots is proposed. Then, on the basis of the entire framework, combined with the simplified virtual force model, the obstacle avoidance problem of the swarm robot in the multi-target search process is solved. Finally, considering the communication interaction problem in the coordination and cooperation of swarm robots and the random line-of-sight problem of individual robots in the actual communication process, the distributed neighborhood interaction model based on a time-varying characteristic swarm with a restricted random line of sight (RS-TVCS) is constructed. By embedding the sub-algorithms in the whole algorithm framework, the MSRCPC algorithm proposed in this paper can greatly improve the search performance of the swarm robot system, making the entire system more scalable and practical.

The remaining parts of this paper are summarized as follows. In section 1, the research background of this algorithm and the research progress at home and abroad was introduced. In section 2, the ideal multi-target search framework for swarm robots is introduced. In section 3, the obstacle avoidance mechanism and distributed communication mechanism of the swarm robot system are described, and the multi-objective search framework of swarm robots considering practical constraints is proposed. The simulation test analysis on the proposed algorithm is conducted in section 4. Finally, the main work is summarized.

## The Framework of Ideal Search Algorithm

In a closed two-dimensional space ***R*^2^**, the task environment for multi-target search of swarm robots can be described by the set {***R***, ***T***, ***S***, ***D***}.where, ***R*** = {***R***_**1**_,…, **R**_***i***_,…, R_***m***_, ***m*** > 1} is the search subject (swarm robots); ***T*** = {***T***_**1**_,…, ***T***_***j***_,…, ***T***_***n***_, ***n*** > ***m***} is the searched target; ***S*** = {***S***_**1**_,…, ***S***_***o***_,…, ***S***_***p***_, ***p*** > 1} is the static obstacle and ***D*** = {***D***_**1**_,…, ***D***_***l***_,…, ***D***_***q***_, ***q*** > ***l***} is the dynamic obstacle. In addition, we let ***w*** exist in the task set {***R***, ***T***, ***S***, ***D***}.

**Figure 1:**
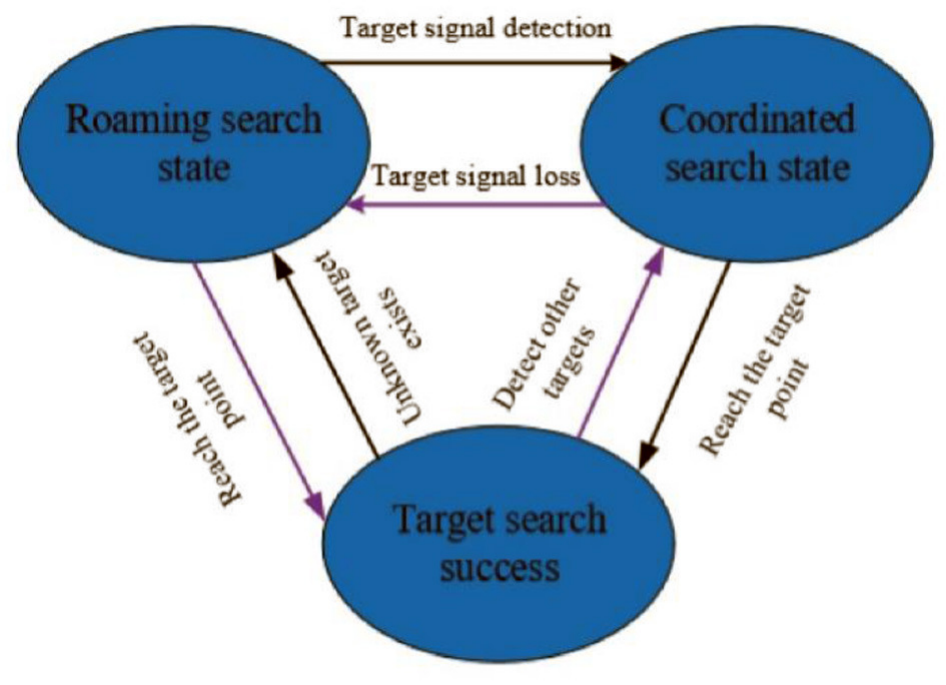
Multi-target search algorithm framework diagram.

The set targets can send out a continuous specific signal, and are randomly distributed in the search map. The sensors carried by swarm robots can detect the strength of the target signal, which cannot determine the direction of the signal. The initial positions of swarm robots are randomly in a certain corner of the search map. In the case without considering obstacles and ideal communication interaction, the multi-target search algorithm framework of swarm robots can be described in the form of a finite state machine. The specific description is shown in Figure 1.

As shown in Figure 1, the basic multi-target search algorithm framework can be described as: when the robot detects the target signal, it enters the coordinated search state, and uses the swarm intelligence optimization algorithm to coordinate the search; when the robot does not detect the target signal, it will follow a certain roaming mechanism to detect the target signal.

### The Multi-Assignment Model Based on Response Threshold

#### Sensor Detection Model

Sensors with different detection distances have different response strengths for target signal, and the function to describe the target signal strength can be set as follows (Manic, 2009):


(1) $$I(i,j)=\{\begin{array}{c} \frac{sQ}{d_{ij}^{2}}+rand(),d_{ij}\leq d_{0}\\ 0,d_{ij}\geq d_{0} \end{array}$$

where Q is the constant power signal sent from the center of the target, ***d***_***ij***_ denotes the distance between the robot and the target, ***d***_**0**_ is the maximum detection distance of the sensor, ***s*** is the signal attenuation factor, ***rand*** is the random disturbance of the signal, and ***I***(***i***, ***j***) is the signal strength between robot and target.

#### Multi-Target Allocation and Design

In the robot roaming search process, the robot may detect multiple target signals. How to make self-organizing decisions on the target signals and find subgroup alliances is the key to the coordinated search of swarm robots. First, the target response function is used to calculate the detection of each target signal for each robot at time ***t***. Then, the probability that the robot selects the target is calculated via the target response signal strength, Finally, the decision about the target based on the roulette probability decision algorithm is made. As shown in Table 1, the induction about the target signal strength of the robot at the moment ***t*** is as follows:

**Table 1:** Detection of target signals by robots members at time *t*.

**Robot**	**Perceived target type**			**Perceive target signal strength**			**Personalized task set**
	***T_**1**_***	***T_**2**_***	***T_**3**_***	***T_**4**_***	***T_**5**_***	***T_**6**_***	
*R_1_*	I-type	Unknown	I-type	0.9358	0	0.3346	{*T_1_,T_3_*}
*R_2_*	Unknown	I-type	Unknown	0	0.6632	0	{*T_2_*}
*R_3_*	Unknown	I-type	Unknown	0	0.6632	0	{*T_2_*}
*R_4_*	Unknown	II-type	I-type	0	0.9358	0.6632	{*T_2_,T_3_*}
*R_5_*	I-type	II-type	I-type	0.3346	0.6632	0.9358	{*T_1_,T_2_,T_3_*}
*R_6_*	Unknown	Unknown	Unknown	0	0	0	none

The probability response process of the ***i***-th robot to the ***j***-th target is:


(2) $$\begin{array}{l} p\left(i,j\right)=\frac{I_{j}^{2}}{\overset{m}{\underset{k=1}{\sum }}I_{k}^{2}} \end{array}$$

where ***I***_***j***_ is the signal strength of the target ***T***_***j***_ detected by the robot ***R***_***i***_. If the robot can detect the number of targets, i.e., m, the probability that ***R***_***i***_ responds to the excitation from target ***T***_***j***_ is ***p***(***i***, ***j***). The ***R***_***i***_ decision-making process of the robot ***R***_***i***_ is as follows:


(3) $$\begin{array}{l} k=\min\left[\overset{m}{\underset{j=1}{\sum }}p\left(i,j\right)\geq rand\left(\right)\right] \end{array}$$

where ***rand* ()** is subject to a uniform score between 0 and 1, and k is the smallest target sequence number satisfying its condition. According to the processed decision-making method, it can be determined from Table 1 that the subgroup alliances composed of the task target set are ***T***_**1**_ = {***R***_**1**_, ***R***_**5**_}, ***T***_**2**_ = {***R***_**2**_, ***R***_**3**_}, and ***T***_**3**_ = {***R***_**4**_}, and the members of ***R***_**6**_ are in the roaming search state and do not participate in the coordinated search.

### The Roaming Search Algorithm Based on Nearest Neighbor Exclusion Diffusion

At the initial moment, the robot cannot detect the target signal. Therefore, it is very important to design an effective individual roaming search model to detect the target signal at the fastest speed. Typical roaming search models include Levy Flight (Viswanathan et al., 1999) and Intermittent Search (Bénichou et al., 2006). However, the roaming search strategies of these models suffer from the following disadvantages: (1) the search efficiency is not high, and (2) the factor of obstacle avoidance is not considered in the search process. Therefore, a new roaming search algorithm, namely, the Nearest Neighbor Exclusion Diffusion (NNED) Algorithm is introduced in this section.

Suppose the position information of the ***i***-th robot in the search space at time ***t*** is expressed as ***X***_***ri***_(***t***) = [***X***_***i***_(***t***), ***Y***_***i***_(***t***)]^***T***^, and the maximum speed of the roaming robot is ***V***_***m***_. The NNED algorithm is described below.

Without considering obstacles, the distance matrix ***D***_***im***_ between the ***i***-th robot and other robots at time ***t*** can be expressed as follows:


(4) $$\begin{array}{l} D_{im}=\left[d_{i1},d_{i2},\ldots ,d_{ik},\ldots ,d_{im}\right] \end{array}$$

where ***d***_***ik***_ is the Euclidean distance between the ***i***-th robot and the ***k***-th robot. Sort equation (4) by row from small to large to obtain the distance sorting matrix ***D***_***is***_.


(5) $$\begin{array}{l} D_{is}=sort\left(D_{im}\right) \end{array}$$

The position sequence information index of the neighboring robot can be expressed as follows:


(6) $$\begin{array}{l} index=find\left(D_{im}\left(1,:\right)==D_{is}\left(1,2\right)\right) \end{array}$$

The repulsion angle **θ** between the ***i***-th robot and the ***index***-th robot is expressed as follows:


(7) $$\theta (t)=\left\{ \begin{array}{l} ac\sin(\frac{X_{i}(t)-X_{index}(t)}{d_{iindex}}),\gamma_{index}(t)\geq \gamma_{i}(t)\\ \pi -ac\sin(\frac{X_{i}(t)-X_{index}(t)}{d_{iindex}}),\gamma_{index}(t)\leq \gamma_{i}(t) \end{array}\right.$$

where ***d***_***iindex***_ is the Euclidean distance between the ***i***-th robot and the ***index***-th robot. Set the expected position of the robot at time ***t*** + 1 as ${X^{\prime }}_{\mathrm{ri}}(t+1)={\left[{x^{\prime }}_{i}(t+1),\;{y^{\prime }}_{i}(t+1)\right]}^{\text{T}}$, and the step size is updated as follows:


(8) $$\left[\begin{array}{c} x_{i}{}^{\prime }(t+1)\\ y_{i}{}^{\prime }(t+1) \end{array}\right]=\left[\begin{array}{c} x_{i}(t)\\ y_{i}(t) \end{array}\right]+\left[\begin{array}{c} V_{m}\cos(\theta (t))\\ V_{m}\sin(\theta (t)) \end{array}\right]$$

Taking into account the boundary constraints, the actual position of the roaming robot is updated as follows:


(9) $$\left\{ \begin{array}{l} V_{x}{}^{\prime }=-V_{m}\cos(\theta (t)),V_{m}\cos(\theta (t))\leq 0\cap x_{i}(t+1)\leq 0\\ V_{x}{}^{\prime }-V_{m}\cos(\theta (t)),V_{m}\cos(\theta (t))\leq 0\cap x_{i}(t+1)\geq L\\ V_{x}{}^{\prime }=-V_{m}\cos(\theta (t)),V_{m}\cos(\theta (t))\geq 0\cup 0\leq x_{i}(t+1)\leq L \end{array}\right.$$

where ***L*** is the search boundary. In the same way, the ***y***-axis velocity component considering the boundary limit can be updated. Set the actual updated position of the robot at time ***t*** + 1 as ***X***_***ri***_**(*t* + 1) = [*x***_***i***_**(*t* + 1)**, ***y***_***i***_**(*t* + 1)]**^***T***^, and the position update of the roaming robot considering boundary constraints is as follows:


(10) $$\left[\begin{array}{c} x_{i}(t+1)\\ y_{i}(t+1) \end{array}\right]=\left[\begin{array}{c} x_{i}(t)\\ y_{i}(t) \end{array}\right]+\left[\begin{array}{c} V_{x}{}^{\prime }\\ V_{y}{}^{\prime } \end{array}\right]\;$$

where ***V***_***ic***_**(*t* + 1) = [*V*′(*x*)**, ***V*′(*y*)]**^***T***^ is update step of the robot roaming speed.

### Coordinated Search Algorithm of Particle Swarm Based on Kinematics Constraints

By analyzing and comparing several benchmark concepts in the cooperative search state of the particle swarm algorithm and swarm robots, it can be found that there is a certain mapping relationship between them. Based on the inertial weight particle swarm algorithm, kinematic constraints can be used to describe this mapping relationship, and the specific expression is as follows (Gudise, 2004):


(11) $$\{\begin{array}{c} V_{ie}(t+1)=\omega V_{Ri}(t)+c_{1}r_{1}(X_{Ri}^{*}(t)-X_{Ri}(t))\\ +c_{2}r_{2}(g_{Ri}^{*}(t)-X_{Ri}(t))\\ V_{Ri}(t+1)=V_{Ri}(t)+(V_{ie}(t+1)-V_{Ri}(t))\cdot \alpha \\ X_{Ri}(t+1)=X_{Ri}(t)+V_{Ri}(t+1)\cdot \delta \\ V_{Ri}(t+1)\leq V_{m} \end{array}$$

where V_ie_(t + 1) is the expected speed of the robot at the next moment, V_Ri_(t + 1) is the speed of the robot at time t, ${\text{X}}_{\text{Ri}}^{*}$(t) is the historical optimal position of the individual robots, g ^*^_Ri_ (t) is the optimal position of the robot at time t, V_Ri_(t + 1) is the actual expected speed considering the kinematics of the robot, X_Ri_(t) is the position coordinate of the robot at time t, X_Ri_(t + 1) is the expected position of the robot at the next time, w is the inertial weight, c_1_ and c_2_ are the individual and social cognitive coefficients of the robot, r_1_ and r_2_ are random numbers uniformly distributed between 0 and 1, α is the inertia coefficient, δ is the step size control factor of the robot, and V_m_ is the limited maximum speed.

Setting the target position as [X_ot_,Y_ot_]^T^, the fitness function of the coordinated search of the robot is as follows:


(12) $$\begin{array}{l} f\left(t\right)=\sqrt{{\left(x_{i}\left(t\right)-X_{ot}\right)}^{2}+{\left(y_{i}\left(t\right)-Y_{ot}\right)}^{2}} \end{array}$$

Because the particle swarm optimization algorithm easily falls into the local best optimum, its inertia weight is improved by combining the actual search situation of the robot in this paper. The basic idea is as follows: when the distance between the particle and the target exceeds a certain threshold, w remains large and the global search is performed; when the distance between the particle and the target is less than a given threshold, w uses its fitness value to performs adaptive non-linear decrement value, fine-grained search and continuously approach the target point. The sigmoid function in the neural network has a strong non-linear approximation ability, whose extreme value ranges between 0 and 1. Since the value of the inertia weight ***w*** in the particle swarm is almost the same, the mapping relationship is as follows:


(13) $$\begin{array}{l} g\left(x\right)=\frac{2}{1+e^{-x}}-1,x≻0 \end{array}$$

Then, a function is introduced into a distance-dependent robots system to adapt the value of inertia weight, and the specific expression is as follows:


(14) $$\omega =\{\begin{array}{c} \frac{2}{1+e^{-5d/d_{l}}}-1,d=f(i)\leq d_{l}\\ 0.8,d=f(i)≻d_{l} \end{array}$$

where ***d***_***l***_ is the set distance threshold and ***d*** = ***f*(*i*)** is the fitness value of the robot.

## The Framework of Swarm Robot Search Algorithm Considering Practical Constraints

On the basis of the ideal multi-target search algorithm framework in the previous section, in this section, the problems of real-time obstacle avoidance and distributed communication in the search process of swarm robots are considered, and a multi-target search algorithm framework for swarm robots considering practical constraints is designed.

### Simplified Virtual Force Model

Aiming at the obstacle avoidance problem in the multi-target search process of swarm robots, introducing a simplified virtual force model can not only perfectly overcome the collision avoidance problem between robots, but also can be well integrated with the entire search algorithm framework, and the performance of the algorithm is also guaranteed.

#### The Construction of Obstacle Avoidance Model

**Figure 2:**
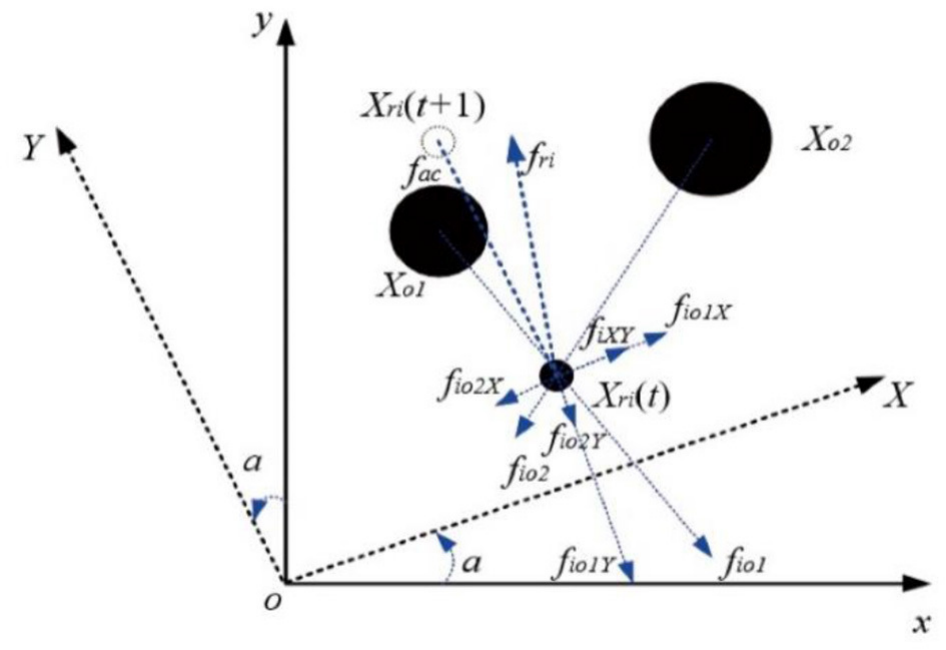
SVFM obstacle avoidance model.

The idea of this model is described in Figure 2. Supposing that the position of the *i*-th robot at time *t* is X_ri_(t), the position of the robot at time *t* + 1 under the framework of the ideal multi-target search algorithm is X_ri_(t + 1). It is obvious from Figure 2 that the local path planned by the robot from *t* to *t* + 1 will coincide with the position of the obstacle.

First, find out the position information of two neighboring obstacles or robots based on the information of obstacles or neighboring robots detected by the sensor of i-th robot sensor, which are X_o1_ and X_o2_ respectively.

Then it is assumed that the robot will be affected by the virtual introduction fac at the next moment and two neighboring obstacles or robot repulsion which are f_io1_ and f_io2_ respectively.

Now define the rotation matrix T_R_ of the new coordinate system XOY generated by rotating the xoy coordinate system counterclockwise by angle a as follows:


(15) $$\begin{array}{l} TR=\left[\begin{array}{cc} \cos\left(a\right) & \sin\left(a\right)\\ -\sin\left(a\right) & \cos\left(a\right) \end{array}\right] \end{array}$$

Set gravity fac as follows:


(16) $$\begin{array}{l} f_{ac}=\left[\begin{array}{c} f_{acx}\left(t\right)\\ f_{acy}\left(t\right) \end{array}\right]=\left[\begin{array}{c} x_{i}\left(t\right)\\ y_{i}\left(t\right) \end{array}\right]-\left[\begin{array}{c} x_{i}\left(t+1\right)\\ y_{i}\left(t+1\right) \end{array}\right] \end{array}$$

The rotation matrix parameter a can be expressed as follows:


(17) $$\begin{array}{l} a=\arctan\left(\frac{f_{acy}\left(t\right)}{f_{acx}\left(t\right)}\right) \end{array}$$

The force function of a given neighbor obstacle or robot is as follows:


(18) $$\begin{array}{l} f_{rep}=k_{1}\cdot {\left(\frac{1}{d_{ik}}-\frac{1}{d_{a}}\right)}^{2} \end{array}$$

where d_a_ is the obstacle avoidance distance of the object (static obstacles, robots, and dynamic obstacles) in the search process, and ***d***_***ik***_ is the distance between the robot and the obstacle in the search process, and k_1_ is the obstacle avoidance parameter of the robot.

Therefore, the coordinate components of obstacles (robots) X_o1_ and X_o2_ to robot i in the XOY coordinate system can be respectively obtained by the simultaneous equations (15)-(18), which are as follows:


(19) $$\begin{array}{l} \left[\begin{array}{c} f_{io1X}\left(t\right)\\ f_{io1Y}\left(t\right) \end{array}\right]=\left[\begin{array}{cc} \cos\left(a\right) & \sin\left(a\right)\\ -\sin\left(a\right) & \cos\left(a\right) \end{array}\right]\cdot \left[\begin{array}{c} f_{io1x}\left(t\right)\\ f_{io1y}\left(t\right) \end{array}\right] \end{array}$$

or


(20) $$\begin{array}{l} \left[\begin{array}{c} f_{io2X}\left(t\right)\\ f_{io2Y}\left(t\right) \end{array}\right]=\left[\begin{array}{cc} \cos\left(a\right) & \sin\left(a\right)\\ -\sin\left(a\right) & \cos\left(a\right) \end{array}\right]\cdot \left[\begin{array}{c} f_{io2x}\left(t\right)\\ f_{io2y}\left(t\right) \end{array}\right] \end{array}$$

where, f_io1x_ and f_io1y_ represent the components of the repulsive force f_io1_ in the coordinate system xoy, and f_io2x_ and f_io2y_ represent the components of the repulsive force f_io2_ in the coordinate system xoy. These components can be all obtained by equation (18).

Finally, only considering the deflection force in the X-axis direction and ignoring the resistance in the Y-axis direction, the final motion direction f_ri_ of the robot is expressed as follows:


(21) $$\{\begin{array}{c} f_{iXY}=f_{io1X}+f_{io2X}\\ f_{ri}=f_{iXY}+f_{ac}\\ v_{ri}(t+1)=v_{if}(t)+v_{ie}(t+1) \end{array}$$

where, v_ri_(t + 1) is the actual speed required by the robot at the next moment, which can be regarded as the vector sum of the robot state update speed v_ie_(t + 1) at time *t* + 1 under the framework of the ideal search algorithm and the deflection speed v_if_ (t) of the neighboring obstacles in the X-axis direction.

#### The Strategy of Robot State Step Update Considering Obstacle Avoidance Constraints

Based on the idea of the SVFM combined with the ideal search algorithm framework with SVFM, the step size update strategy of the robot in different search states is given in this section. When the robot is in the roaming search state, r_state_ = 0; when the robot is in the coordinated search state, r_state_ = 1. The speed step update strategy of swarm robots in different states is as follows:


(22) $$\{\begin{array}{c} v_{ri}(t+1)=V_{ic}(t+1),r_{state}=0\cap d_{ij}≻d_{a}\\ v_{ri}(t+1)=V_{ie}(t+1),r_{state}=1\cap d_{ij}≻d_{a}\\ v_{ri}(t+1)=V_{ic}(t+1)+v_{if}(t),r_{state}=0\cap d_{ij}\leq d_{a}\\ v_{ri}(t+1)=V_{ie}(t+1)+v_{if}(t),r_{state}=1\cap d_{ij}\leq d_{a} \end{array}$$

The position update strategy of swarm robots considering obstacle avoidance constraints is as follows:


(23) $$\begin{array}{l} x_{ri}^{*}\left(t+1\right)=x_{ri}\left(t\right)+v_{ri}\left(t+1\right) \end{array}$$

### The Distributed Neighborhood Communication Mechanism Based on Time-Varying Characteristic 179 Swarm With Restricted Random Line of Sight (RS-TVCS)

#### The Communication Model Based on RS-TVCS

In biological research, perception and communication between animal groups are often limited by perception distance. For example, when the Ouqiong bird population flies in formation, its individuals can only exchange information with neighboring individuals within its communication radius to form a local communication network. There is a common neighboring individual between two individuals, and they cannot directly communicate and interact. Through sharing the information of common neighboring individuals, it can spread to the individuals outside their neighbors to form a global communication network. Based on this idea, a representation based on distributed neighborhood communication is defined. The communication-based neighborhood of robot i is a set of teammates within a fixed radius dc to the position of robot i, which can be written as (Xue et al., 2009):


(24) $$\begin{array}{l} \Omega \left(r_{i}\right)=\left\{r_{j\in m,j\neq i},||x_{ri}-x_{rj}||\leq d_{c}\right\} \end{array}$$

where Ω is the communication-based neighborhood, m is the number of members in the swarm, and r_i_ denotes the robot i. x_ri_ and x_rj_ are the spatial positions of robots i and j, robots respectively. d_c_ is the maximum communication radius.

During swarm moving, the neighborhoods may change over time, causing the whole swarm to be divided into several dynamically changing sub-swarms. Xue et al. defined those sub-swarms with the concept of Time-Varying Characteristic Swarm (TVCS). The TVCS of robot i at time *t* can be represented as follows (Junior and Nedjah, 2016):


(25) $$\begin{array}{l} \Omega \left(r_{i}\right)\left(t\right)=r_{i}\cup \left\{r_{j\in m,j\neq i},||x_{ri}\left(t\right)-x_{rj}\left(t\right)||\leq d_{c}\right\} \end{array}$$

where Ω(r_i_)(t) represents the TVCS of robot i. The number of members in a TVCS is dynamically changing, i.e., ri can only able to communicate with other agents in Ω(r_i_)(t) at the time *t*. Taking into account the limited field of view in the robot signal interaction process, Yang et al. (2019) defined a notation of visual limited TVCS (V-TVCS), which can be written as:


(26) $$\begin{array}{l} \Omega_{v}\left(r_{i}\right)\left(t\right)=r_{i}\cup \left\{r_{j\in m,j\neq i},||x_{ri}\left(t\right)-x_{rj}\left(t\right)||\leq d_{c}\wedge \varphi_{i,j}\leq \frac{\omega }{2}\right\} \end{array}$$

where Ω_v_(r_i_)(t) is the V-TVCS. ω is the single of view of i-th robot, and its sight range is generally set to ϕ_i,j_ ϵ (0, 2π]. ϕ_i,j_ is the sight judgment vector of robots i and j, which is expressed as follows:


(27) $$\begin{array}{l} \varphi_{i,j}=\langle r_{ij}\left(t\right),v_{ri}\left(t\right)\rangle \end{array}$$

where, r_ij_(t) is the location vector of robots i and j, v_ri_(t) is the speed vector of the ith robot, and 〈 r_ij_(t), v_ri_(t)〉 is the angle between vectors r_ij_(t) and v_ri_(t).

**Figure 3:**
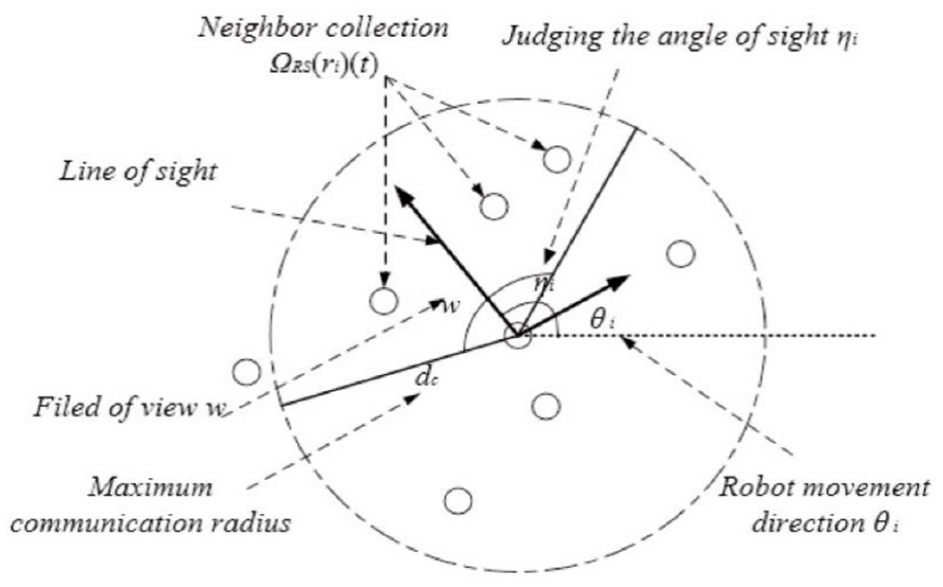
Schematic diagram of individual neighbor collection.

Since the line of sight of the robot is not always in the direction of its speed in the process of motion, it is assumed that the line of sight of individual robots changes randomly along the direction of movement in this paper and that the change law obeys the normal distribution, namely, η ~ N(0, σ^2^), where σ is the standard deviation of the deflection angle of the line of sight, and the mean value is 0, indicating that the probability of the individual going straight ahead is greater than that of information interaction to the diagonal side. Considering the limitation of the random line of sight of the robot, the relationship structure diagram of the neighborhood distributed neighborhood communication based on RS-TVCS designed in this paper is shown in Figure 3.

The distributed neighborhood communication mechanism based on RS-TVCS is defined as followed:


(28) $$\begin{array}{l} \Omega_{RS}\left(r_{i}\right)\left(t\right)=r_{i}\cup \left\{r_{j\in m,j\neq i},||x_{ri}\left(t\right)-x_{rj}\left(t\right)||\leq d_{c}\wedge \varphi_{i,j}\leq \frac{\omega }{2}\right\} \end{array}$$

where, the expression of ϕ_i,j_ is as follows:


(29) $$\begin{array}{l} \varphi_{i,j}=\langle r_{ij}\left(t\right),LOS_{ri}\left(t\right)\rangle \end{array}$$

where LOS_ri_(t) is the vector of line of sight. When LOS_ri_(t) = v_ri_(t), it indicates that the line of sight of the robot is consistent with its moving direction. Therefore, the V-TVCS distributed communication mechanism based on V-TVCS is only a special case of RS-TVCS. RS-TVCS has better scalability and practicability than V-TVCS.

#### RS-TVCS Distributed Network Connected Subset Judgment Based on BFS Algorithm

The global communication network based on the RS-TVCS will change with the dynamic migration of swarms. Under the ideal search algorithm framework, it will iteratively change with the position of the robots, which will make it impossible for some robots to interact with each other, thus forming connected subgroups. Therefore, based on graph theory, assuming that the position of each robot at a certain moment represents a dynamic node, the connected subgroup of each robot is determined based on the idea of the breadth first search (BFS) algorithm. Through this algorithm, the interactive information of each robot under the entire global communication network based on RS-TVCS can be obtained, so as to realize the coordinated search of swarm robots.

The specific ideas are as follows:

1) Taking the position of the robot at time *t* as the node, the weight matrix d_ij_ is constructed by using the distance between the two points as follows:
  (30) $$\begin{array}{l} d_{i,j}=\left[\begin{array}{cccccc} 0 & d_{1,2} & \cdots & d_{1,j} & \cdots & d_{1,m}\\ \vdots & 0 & \vdots & \vdots & \vdots & \vdots \\ d_{m,1} & d_{m,2} & \cdots & d_{m,j} & \cdots & 0 \end{array}\right] \end{array}$$
2) Through the neighborhood judgment conditions of equations (28) and (29), the neighborhood weight matrix is constructed. When the neighborhood judgment conditions are not satisfied between the robots i and j, the weight between the two robots (nodes) is 0; otherwise, the weight between the two robots (nodes) is Euclidean distance value.
3) Based on the idea of the BFS (Awerbuch and Gallager, 1987; Jia et al., 2008; Wang et al., 2020) algorithm, all the connected nodes of the neighborhood weight matrix are found to obtain the neighborhood communication information of each robot in the global network.

#### The Flow of Multi-Target Search Algorithm Swarm Robots Considering Practical Constraints

**Figure 4:**
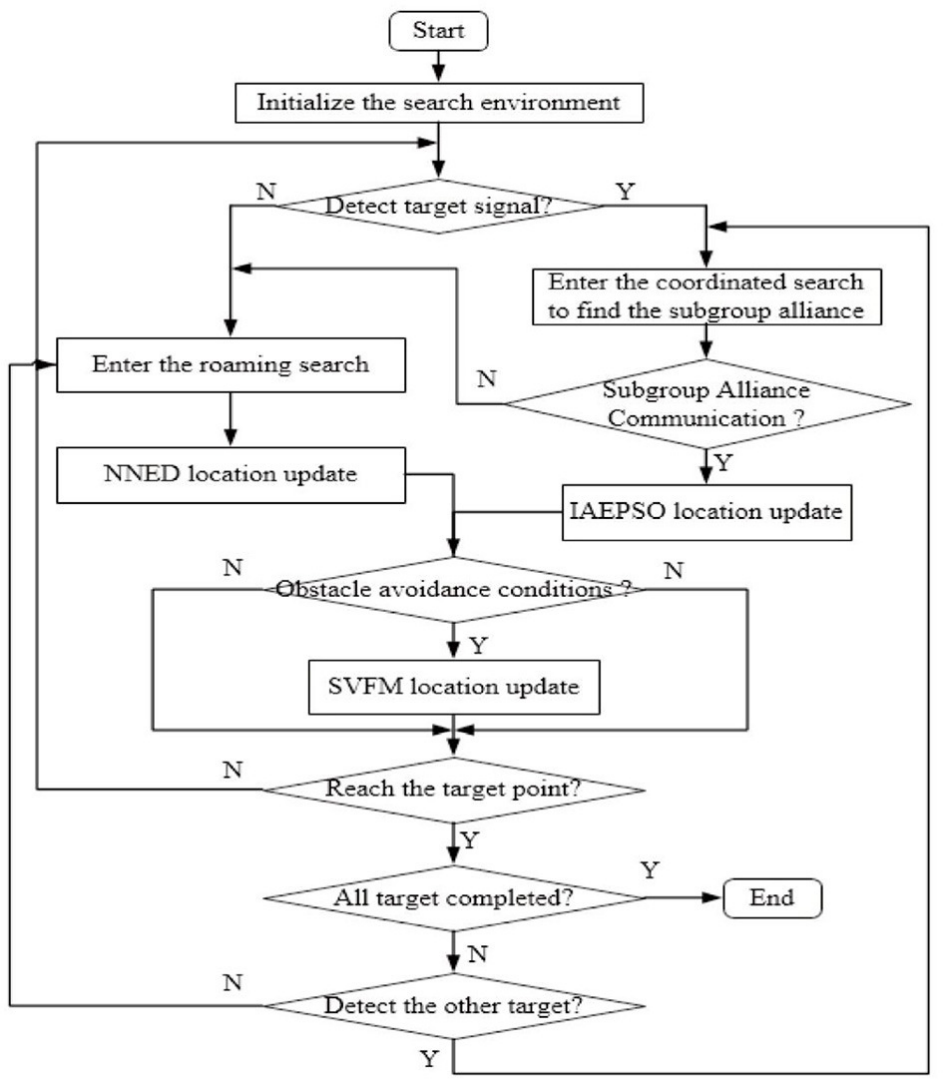
MSRCPC algorithm flow diagram.

Under the ideal multi-target search algorithm framework, the distributed communication problem in the search environment is combined with the real-time obstacle avoidance problem. The flow chart of the multi-target search algorithm for swarm robots considering practical constraints (i.e., MSRCPC) is shown in Figure 4.

The main sub-algorithms involved in the proposed algorithm include NNED roaming search algorithm, IAEPSO coordinated search algorithm, TRT multi-target task assignment, obstacle avoidance algorithm based on SVFM, and distributed communication algorithm based on RS-TVCS. The entire algorithm framework basically considers all the problems in the search process of swarm robots, which greatly enhances the scalability and usability of the algorithm.

## Simulation

In this section, the proposed MSRCPC algorithm has been verified by several experiments in Matlab2019a. First, the MSRCPC algorithm is described in detail by simulating the search behavior of swarm robots in single-target environments and multi-target environments. Then, four multi-target search comparison modes are set up, and the simulation tests are carried out 30 times by using different modes simulation tests 30 times under different group sizes. The effectiveness of the MSRCPC algorithm is verified by comparing and analyzing the simulation results.

### The MSRCPC Algorithm Test

In this part, the basic parameter settings of the MSRCPC algorithm are shown in Table 2.

**Table 2:** The table of MSRCPC algorithm parameter.

**Symbol**	**Symbolic meaning**	**Parameter value**
*m*	Swarm robotics	10–100
*n*	Search target	1–10
*s*	Signal attenuation factor	0.1
*Q*	Constant power signal	10,000
*d0*	Sensor maximum detection distance	100
*Vm*	Robot maximum speed	10
α	Inertia coefficient	0.4
δ	Step size control factor	0.6
*dl*	Adapted distance threshold	100
*k1*	Obstacle avoidance parameter	0.8
*da*	Obstacle avoidance distance	80
*dc*	Neighborhood communication distance	100
*w*	Robot sight range	150

With constant basic parameters, the algorithm is applied to single-target and multi-target simulation environments. In view of the randomness of the algorithm, an algorithm search process is randomly recorded to describe the search mechanism and show the performance of the algorithm in detail.

#### The Single-Target Search Test in Unknown Complex Environments

**Figure 5:**
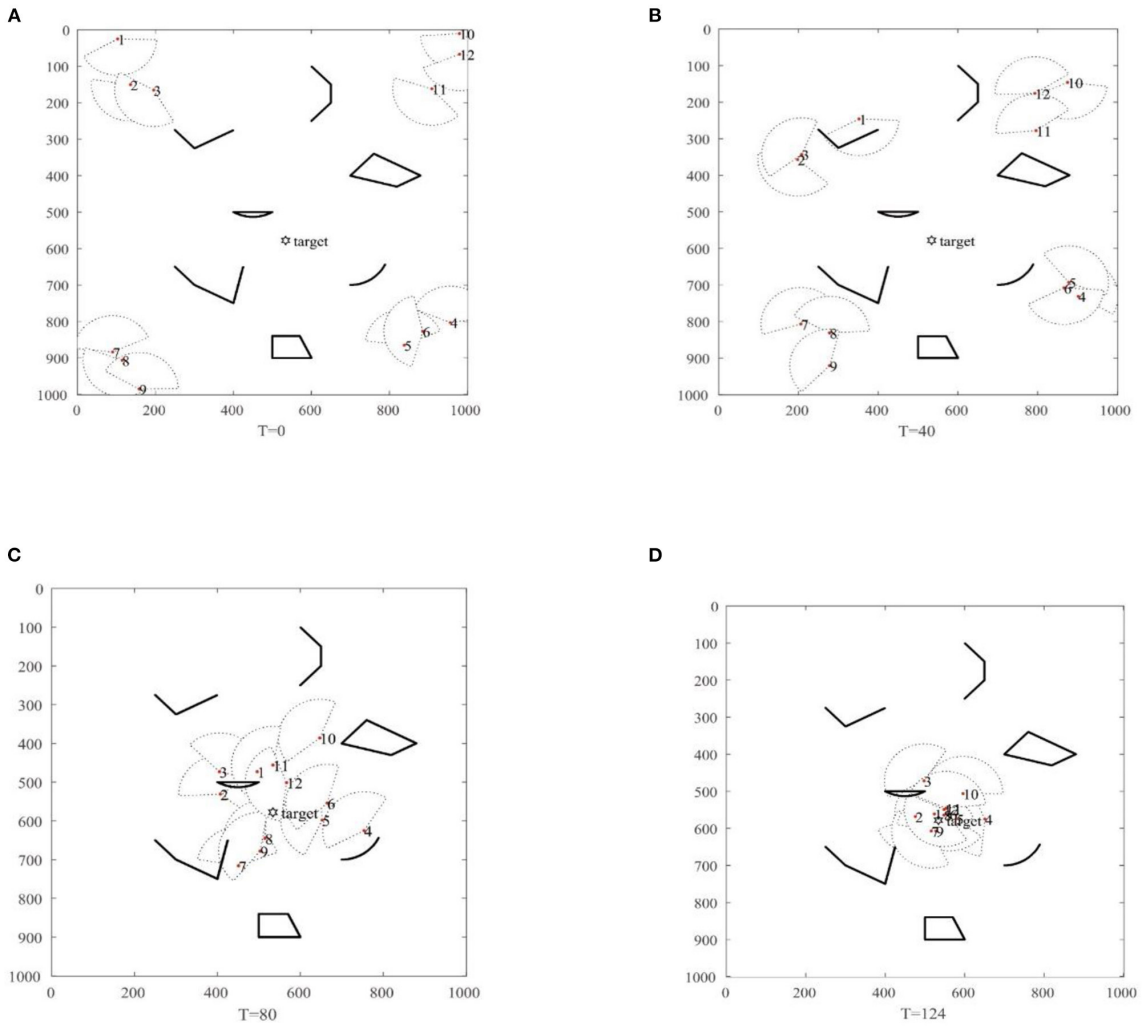
The figure of MSRCPC single-target search. **(A)** T = 0, **(B)** T = 40, **(C)** T = 80, **(D)** T = 124.

The initial environment settings of the swarm robotics single-target search simulation for swarm robots are shown in Figure 5A. As shown in Figure 5A, at *T* = 0, swarm robots are distributed in the corners of the search space, represented by red dots. The position of the target to be searched is set in the middle of the search space, represented by a black regular hexagon. The various black shapes in the figure represent obstacles in the search space. For the robot, the maximum speed is 10, the direction of its initial speed is random, the communication range is limited to 150 degrees, and the direction of moving speed is inconsistent with the direction of the line of sight, and meets the communication conditions of robot in RS-TVCS. Since the robot does not detect the target signal at the initial moment, the NNED algorithm is used to perform random search and diffusion. When *T* = 40, the robot still does not detect the target signal point, and the NNED algorithm continues to be used to randomize, as shown in Figure 5B. As shown in Figure 5C, at *T* = 80, the No. 1 robot detects the target signal, and then based on the RS-TVCS algorithm proposed in this paper, the number of the robots is learned that can communicate, and the group communication is conducted to form sub swarms. The robot that can detect the target signal through group information sharing switches from the roaming search state to the coordinated search state, and uses the IAEPSO algorithm to coordinate the search for the target point. Finally, as shown in Figure 5D, at *T* = 128, the robots numbered 5, 6, 7, 8, 9, 11, and 12 basically converge to the target point, and the target search is successful. The simulation search process with the MSRCPC algorithm can basically be divided into two stages: roaming search and coordinated search.

**Figure 6:**
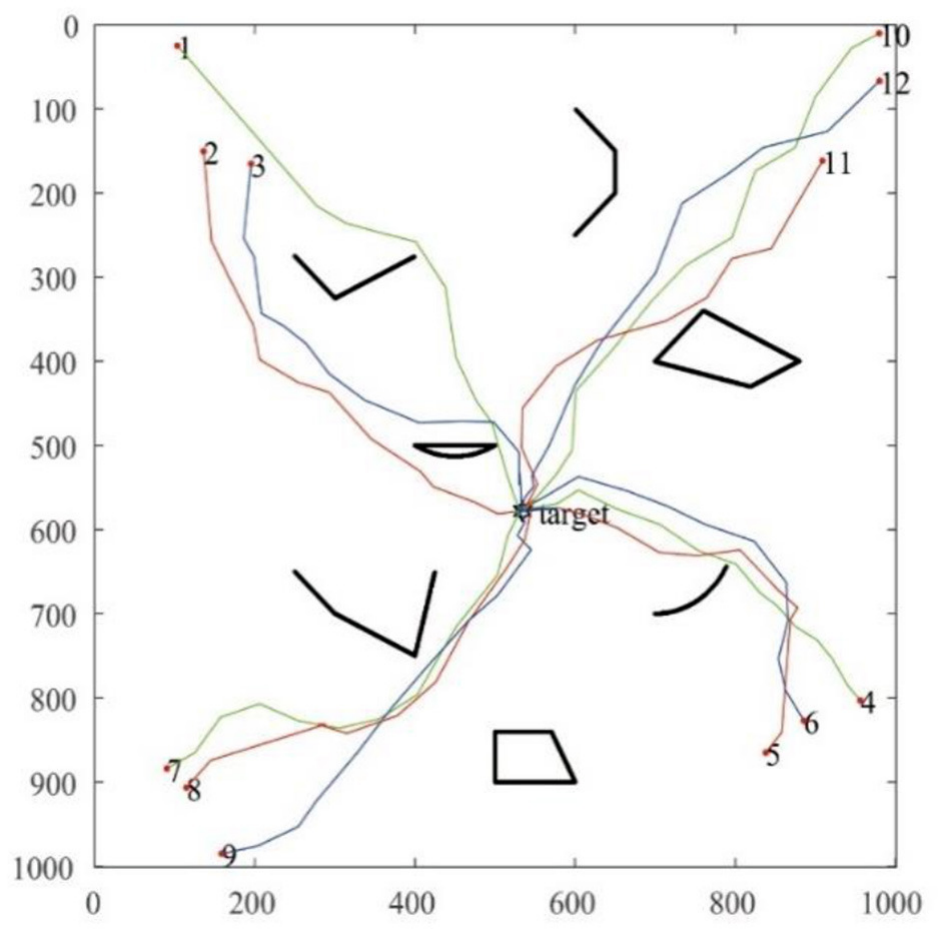
MSRCPC single-target search path simulation diagram.

The search path of the robot recorded in this single-target simulation is shown in Figure 6, and it can be seen that the MSRCPC algorithm can not only search for targets quickly and accurately, but also can intelligently avoid obstacles, and has good cluster avoidance performance.

#### The Multi-Target Search Test in Unknown Complex Environments

**Figure 7:**
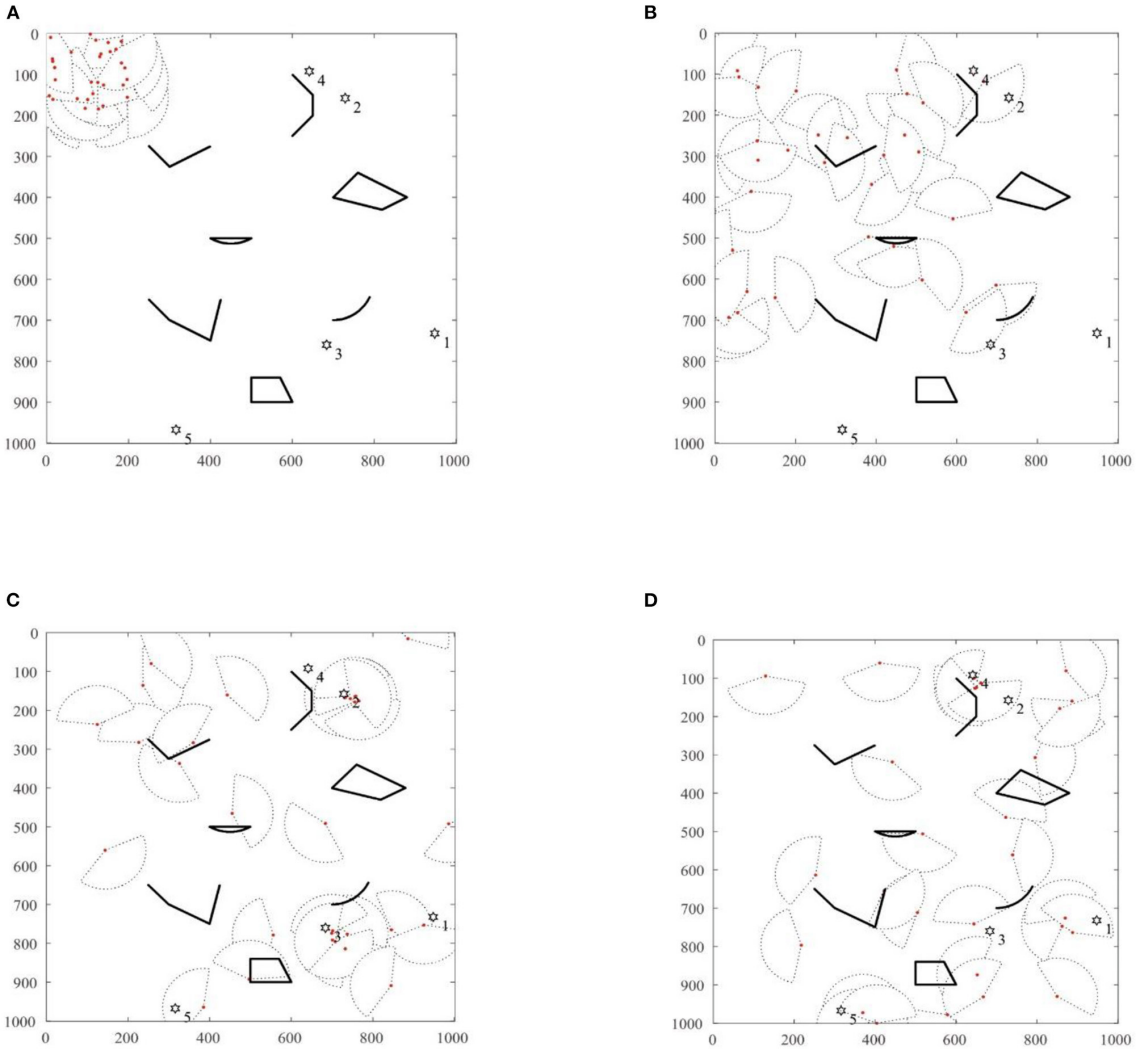
The figure of MSRCPC multi-target search. **(A)** T = 0, **(B)** T = 87, **(C)** T = 123, **(D)** T = 186.

Given that the initial number of robots is 30 and the number of targets is 5, other algorithm parameters are consistent with those of the single-target search algorithm in the previous section. The specific simulation search process is shown in Figures 7A–D.

In Figure 7A, at *T* = 0, the drone swarm is randomly distributed at 200 × 200 unit positions in the search space, and the target points are randomly distributed in the 1,000 × 1,000 search space. The black irregular shape represents the obstacles in the search environment, and the proposed RS-TVCS method is adopted by the robot group to communicate. Based on the RS-TVCS communication rules, using the BFS algorithm, it can be known that at *T* = 0, the 30 robots are neighbors and can maintain information sharing. As shown in Figure 1, the 30 fan-shaped shared areas of the robots are group global communication area of the robot group. The robot does not detect the target signal in the global communication area, and the robot is in a roaming search state, that is, it uses the NNED algorithm to perform a roaming search at its maximum speed.

In Figure 7B, when *T* = 87, some robots detect the No. 2 target signal and the No. 3 target signal. At this time, the robots in the RS-TVCS global communication neighborhood share local information, and then perform target assignment based on the TRT model to form a subgroup alliance and enter the coordinated search state. However, the robots that fail to communicate with their subgroups continue to maintain the roaming search state and perform roaming searches. In addition, the No. 4 robot detects the No. 2 target signal and the No. 13 robot detects the No. 3 target signal. Based on the RS-TVCS neighborhood communication algorithm, it can be seen that the robots 6, 8, 9, 11, 17, 21, 24, and 29 that share information with the No. 4 robot form a subgroup alliance. Their state changes to the coordinated search state, and then a collaborative search will be conducted on the No. 3 target. In the same way, the No. 29 robot that shares information with the No. 13 robot forms a subgroup alliance, and then performs an accurate collaborative search on the No. 3 target. Since the remaining robots cannot communicate with the two subgroup alliances, or detect the target signal, they continue to maintain the roaming search state for random diffusion.

As shown in Figure 7C, when the MSRCPC algorithm iterate to *T* = 123, the subgroup alliances that perform a coordinated search on the targets No. 2 and No. 3 converge to the vicinity of targets No. 2 and No. 3, respectively, and the search for targets No. 2 and No. 3 succeeds. At the same time, the search target information disappears, and the subgroup alliance is disbanded. The formation of robot is the No. 9 robot and the No. 26 robot detect the signal of the No. 1 target and the No. 5 target, respectively. Similarly, according to the solution of the RS-TVCS distributed communication model, it can be seen that 8 robots (2, 18, etc.), which can share information with the No. 9 robot form a subgroup alliance to conduct a collaborative search for the No. 1 target, while the No. 26 robot that fails to interact with other robots cannot obtain communication and maintains a coordinated search alone. In addition, the remaining individual robots that fail to communicate with the target groups No. 1 and No. 5 continue to roam and search using the NNED algorithm.

Finally, as shown in Figure 7D, at *T* = 186, the robots successfully detect the No. 4 target, and the search of swarm robots ends.

#### The Simulation Analysis of the MSRCPC Algorithm

In the test of the single-target and multi-target search process, the MSRCPC algorithm proposed in this paper has the following advantages. (1) The search process of the algorithm mainly includes roaming search processes and coordinated search processes. In the roaming search process, the robot cannot obtain the prior information of the target, and spreads the search space at the fastest speed; in the coordinated search process, by obtaining the target information, the robots are determined by the RS-TVCS communication interaction model in the global communication range, and then the sub-group alliances approach the optimal position of the target point based on the group optimal information and individual optimal information in the IAEPSO algorithm. (2) Self-organization and adaptability are embodied in the process of the target search of swarm robots. In the process of target searching, swarm robots adaptively transform their own state by acquiring information of the external environment or sharing local information and participating in task collaboration. (3) The intelligence of swarm robots in the target search process is also reflected. In the process of roaming search and coordinated search, individual robots can realize intelligent obstacle avoidance by sensing the information of the external environment and successfully avoiding obstacles. In order to verify the performance of the MSRCPC algorithm, a series of comparative experiments are carried out in the next part.

### Comparison and Discussion of MSRCPC Algorithm Simulation

In this part, the four sets of comparison modes are set up to further verify the superiority of the MSRCPC algorithm based on the multi-target search framework of the finite state machine. The settings of the four comparative search models are shown in Table 3.

**Table 3:** The four search algorithm mode table.

**Mode**	**Task allocation**	**Roaming search**	**Coordinated search**	**Obstacle avoidance**	**Distributed communication**
Mode1	ITRT	NNED	EPSO	SVFM	v–TVCS
Mode2	ITRT	NNED	IABA	SVFM	v-TVCS
Mode3	ITRT	NNED	IAEPSO	SVFM	v-TVCS
Mode4	ITRT	NNED	IAEPSO	SVFM	RS-TVCS

Based on its framework, the search algorithm is divided into the following five parts, namely, multi-target task allocation model, roaming search algorithm, coordinated search algorithm, cluster obstacle avoidance, and distributed communication model.

For Mode 1, the NNED algorithm is adopted for roaming search, the traditional TRT model is used to assign tasks to the target, the EPSO algorithm proposed in Gudise (2004) is applied to coordinated search, the 2D-SVFM model (Xinjie, 2020) is applied to group obstacle avoidance, and the V-TVCS model proposed in Yang et al. (2019) is used for robot communication. For Mode 2, the IABA algorithm proposed in Tang et al. (2020) is applied to the robot coordinated search, and the other sub-algorithms remain constant. For Mode 3, the proposed IAEPSO algorithm is applied to the robot coordinated search, and the remaining sub-algorithms remain unchanged. For Mode 4, the proposed MSRCPC algorithm is used to set up the search experiment.

**Figure 8:**
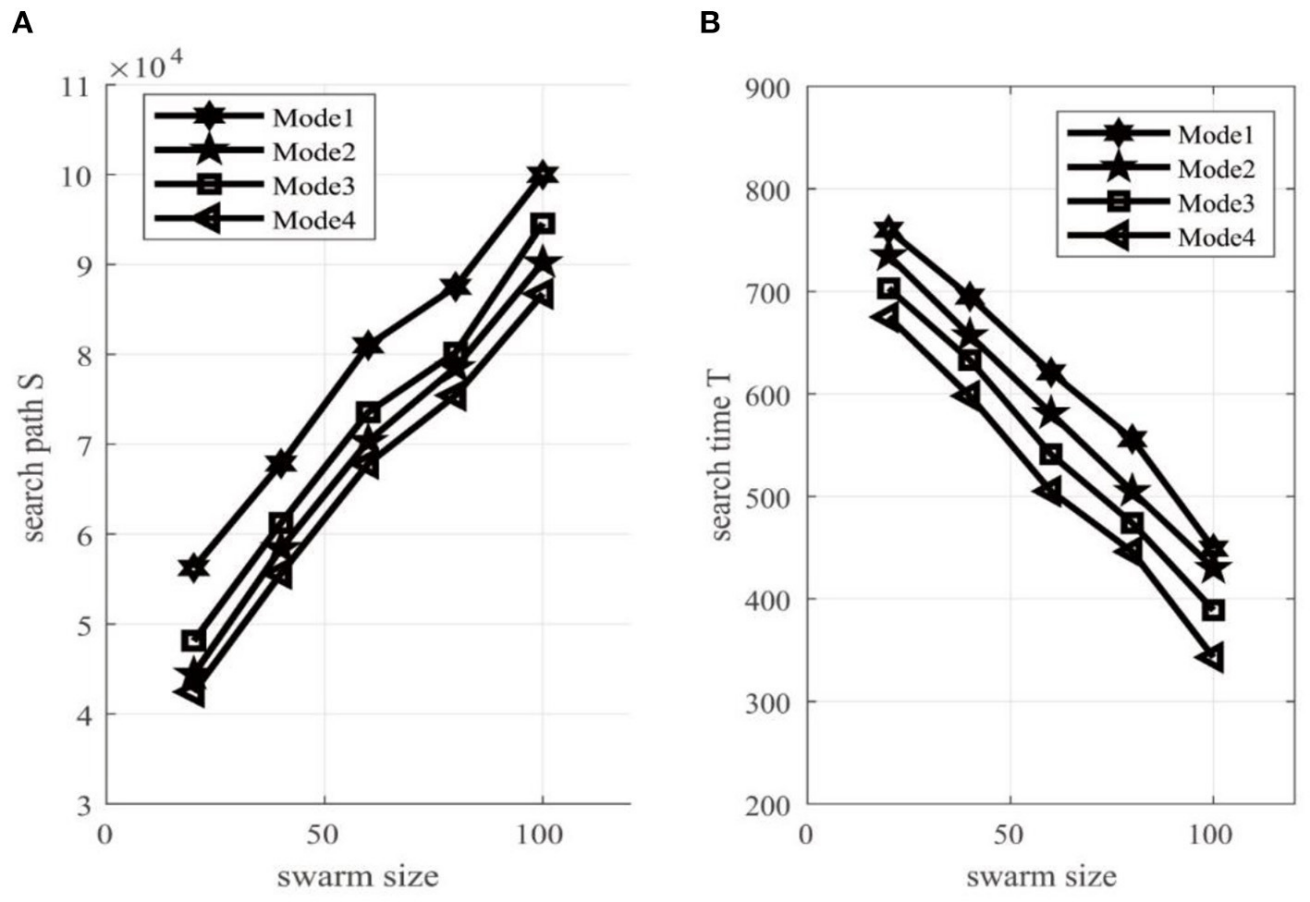
**(A)** Searching time *T* and **(B)** total energy consumption S of the swarm robotics system.

When the number of targets in the search environment of swarm robots is 10, by changing the number of swarm robots, these four modes were used to conduct 30 simulation search experiments. The change of the search path S and the mean value of the search time *T* of the swarm robots with the population number is shown in Figure 8; Table 4.

**Table 4:** System performance comparison statistics table of four search modes.

**Swarm size**	**Search**	***T***			***S***		
	**model**	**Max**	**Mean**	**Min**	**Max**	**Mean**	**Min**
20	Mode1	845	771.234	765	6.091E + 04	5.876E + 04	5.491E + 04
	Mode2	786	763.541	698	4.871E + 04	4.791E + 04	4.469E + 04
	Mode3	754	737.421	712	4.452E + 04	4.912E + 04	5.367E + 04
	Mode4	707	675.741	674	4.087E + 04	4.178E + 04	4.619E + 04
40	Mode1	746	698.39	678	7.189E + 04	6.654E + 04	6.291E + 04
	Mode2	645	645.48	610	5.908E + 04	5.769E + 04	5.491E + 04
	Mode3	631	654.31	631	6.598E + 04	6.235E + 04	5.561E + 04
	Mode4	607	587.431	571	5.798E + 04	5.668E + 04	5.247E + 04
60	Mode1	639	619.361	591	8.271E + 04	8.018E + 04	7.789E + 04
	Mode2	579	550.189	547	6.154E + 04	5.789E + 04	5.554E + 04
	Mode3	619	576.981	539	7.981E + 04	7.467E + 04	7.086E + 04
	Mode4	520	504.861	476	7.234E + 04	6.967E + 04	6.431E + 04
80	Mode1	581	543.187	538	8.913E + 04	8.761E + 04	8.531E + 04
	Mode2	489	471.67	468	8.318E + 04	7.971E + 04	7.689E + 04
	Mode3	549	518.60	471	8.241E + 04	7.987E + 04	7.618E + 04
	Mode4	459	449.356	423	8.089E + 04	7.709E + 04	7.136E + 04
100	Mode1	471	451.61	406	1.109E + 05	9.971E + 04	9.012E + 04
	Mode2	389	368.071	319	1.012E + 04	9.456E + 04	8.956E + 04
	Mode3	397	369.178	365	9.780E + 04	9.438E + 04	9.129E + 04
	Mode4	368	326.678	306	9.109E + 04	8.754E + 04	8.497E + 04

It can be seen from Figure 8; Table 4 that when the number of constant search targets is 10, as the scale of the swarm robots increases, the search time of the swarm robot system will decrease, but the system energy consumption of the swarm robots will increase. Therefore, we are surprised to find that how to balance the search time and energy consumption of the entire system by balancing the scale of swarm robots is a basic problem in the practical application of swarm robot systems.

By comparing Mode 3 with Mode 1 and Mode 2, it can be seen that in the entire multi-target search framework, the proposed IAEPSO coordinated search algorithm has better performance than EPSO and IABA algorithms in different population sizes. The main reason is that the adaptive inertia weight set by the IAEPSO algorithm can satisfy the coordinated search behavior of the robot. However, when the target signal exceeds a certain threshold, the robot has a larger inertia weight and can conduct a large-scale coordinated search. When the target signal is less than a certain threshold, in order to avoid the robot oscillating around the target, the algorithm can adaptively adjust the motion behavior of the robot to avoid the oscillation of the path, thereby reducing system energy consumption.

From the performance comparison curves of Mode 4 and Mode 3, it can be seen that in the case of the other sub-algorithms being the same, the search performance of swarm robots using the RS-TVCS distributed communication algorithm is better than that of Mode 3. The main reason is that the RS-TVCS distributed neighborhood communication model can meet the communication interaction performance of actual swarm robots. Using the RS-TVCS model in the process of forming subgroup alliances will make the configuration of the robot members within each member more reasonable, which can greatly improve the utilization of robot members and provide a more efficient search for the entire algorithm framework.

All in all, compared with the first three modes, the search performance of the swarm robotics can be improved by at least 25 by using the proposed MSRCPC algorithm (Mode 4).

## Conclusion

The multi-target search problem of swarm robots in unknown complex environments is studied in this paper. The main innovations are as follows. (1) Aiming at the target search problem of swarm robots in actual environments, a target search framework based on a finite state machine is proposed. The proposed framework can not only solve the single-target search problem, but also solve the multi-target search problem, which improves the applicability of this algorithm in actual search scenarios. (2) In this algorithm, the problem of cluster obstacle avoidance is considered as a problem in the actual search environment, and the intelligence of cluster search for the robot is reflected. (3) In order to solve the distributed communication interaction problem in the unknown environments, by considering the random communication between individual robots and the limited visual area, a RS-TVCS model is proposed, which overcomes the shortcomings of the V-TVCS communication model.

Simulation analysis and comparison experiments show that this proposed algorithm has good search performance and strong scalability and stability, and can adapt to any search environment. In addition, we find, surprisingly, that the balance of search performance of the swarm robot system is related to the number of swarm robots. Therefore, how to balance the search path and search time of swarm robot systems by setting a certain number of swarm robots is the focus of further research.

## Data Availability Statement

The original contributions presented in the study are included in the article/supplementary material, further inquiries can be directed to the corresponding author/s.

## Author Contributions

YZ: conceptualization, methodology, and writing—original draft preparation. AC: methodology, software, investigation, and writing—reviewing and editing. XH and XB: software and writing—reviewing and editing. All authors contributed to the article and approved the submitted version.

## Funding

This work was supported in part by the National Defense Basic Research Program of China under Grant JCKY2019403D006, the Outstanding Youth Project of the Education Department of Hunan Province of China under Grant 19B200, the Doctoral Scientific Research Initial Funds of the Human University of Science and Technology under Grant E56126, and the Special Project of Engineering Research Center (Item No: Lgy18gz006).

## Conflict of Interest

The authors declare that the research was conducted in the absence of any commercial or financial relationships that could be construed as a potential conflict of interest.

## Publisher's Note

All claims expressed in this article are solely those of the authors and do not necessarily represent those of their affiliated organizations, or those of the publisher, the editors and the reviewers. Any product that may be evaluated in this article, or claim that may be made by its manufacturer, is not guaranteed or endorsed by the publisher.
